# Supplementary material for: WeChatting for Health: What Motivates Older Adult Engagement with Health Information
Source: Healthcare (Basel). 2021 Jun 18;9(6):751. doi: 10.3390/healthcare9060751 (PMC8234300; doi:10.3390/healthcare9060751)
Supplement: Supplementary file 1 [file healthcare-09-00751-s001.zip › healthcare-1226542-supplementary.pdf]

Table S1. Older adult's motivations for health purpose on WeChat.

Motivations	Frequency	Motivations	Frequency
I have acquired all kinds of medical knowledge and healthcare information through WeChat, which is difficult for me to obtain from other channels	20	To express personal stance on health issues makes me feel satisfied	10
I can obtain emotional and informational support from others on WeChat when discussing some health issues and personal health conditions	17	It is convenient and time-saving to use WeChat to book online medical registration	9
WeChat helps me to access tailored and customized healthcare advice from online disease diagnosis and treatment	15	WeChat offers online medical diagnosis and telehealth, which helps me quickly find famous doctors I would normally struggle to get an appointment with	9
I can get more health education on WeChat	15	To filter health-related misinformation and offer high quality information for WeChat friends and groups	8
WeChat helps me to access tailored and customized healthcare advice from online disease diagnosis and treatment	14	WeChat allows me to monitor and filter health-related rumors and fake news	7
I often share my experiences and knowledge on medication, daily care, diet and exercise with people with similar health conditions on WeChat	14	My community uses WeChat to issue health notifications such as vaccinations and free clinic information	7
To show my care to my family and friends by sharing meaningful health information on WeChat	13	It is very efficient and convenient for the community to deliver health notifications through WeChat	7
To build and consolidate relationships by sharing important health information	13	I feel empowered to sharing health information and to give health-related advice to WeChat friends and groups	6
Through the WeChat official account and Moments, I can keep abreast of the latest developments in public health events	12	I often ask my friends to exercise together through WeChat, which improved my enthusiasm	6
WeChat provides timely and authoritative coverage of public health issues and health policy	11	To provide professional drug information	3
I can search for any health information I need from WeChat	11	WeChat sports and health code help me manage my health	3

Table S2. Demographic information of participants (n = 690).

Measure	Range	M (SD)	Percentage (%)
Gender	Female (vs. male)		47.5
Age in years	50–74	60.47 (6.21)	
Education	1–5 (from primary school or below to Master's degree or above)	2.85 (0.89)	
Monthly income	1–5 (from CNY 1000 or below to CNY 7000 or above)	2.91 (1.07)	
Health status	1–5 (very bad to very good)	2.53 (0.935)	

Table S3. Factor analysis of motivations for health-purpose on WeChat.

Motivation Items	Factors							
	1	2	3	4	5	6	*M*	*SD*
**Social Support**								
I can obtain emotional and informational support from others on WeChat when discussing health issues and personal health conditions	**0.765**	0.125	0.039	0.166	0.141	0.088		
I often ask my friends to exercise together through WeChat, which improved my enthusiasm	**0.732**	0.221	0.104	0.14	0.136	0.135		
I often share my experiences and knowledge on medication, daily care, diet, and exercise with people with similar health conditions on WeChat	**0.731**	0.114	0.036	0.183	0.248	0.075	3.8	0.80
I am a member of some health and wellness groups. When we talked about health-related topics, I felt the emotional support and comfort from other group members	**0.729**	0.216	0.127	0.138	0.046	0.007		
My community uses WeChat to issue health notifications such as vaccinations and free clinic information	**0.699**	0.185	0.115	0.138	0.099	0.089		
**Information Need**								
I can get more health education on WeChat	0.219	**0.788**	0.145	0.103	0.158	0.035		
WeChat helps me to access tailored and customized healthcare advice from online diseases diagnosis and treatment	0.152	**0.752**	0.092	0.106	0.138	0.113		
I have acquired all kinds of medical knowledge and healthcare information through WeChat, which is difficult for me to obtain from other channels	0.197	**0.747**	0.051	0.172	0.181	0.095	3.66	0.83
I can search for any health information I need from WeChat	0.221	**0.727**	0.103	0.128	0.158	0.12		
**Technological Convenience**								
WeChat offers online medical diagnosis and telehealth, which helps me quickly find famous doctors I would normally struggle to get an appointment with	0.12	0.062	**0.897**	0.05	0.162	0.108		
It is convenient and time-saving to use WeChat to book online medical registration	0.102	0.123	**0.839**	0.063	0.187	0.154	3.88	0.88
It is very efficient and convenient for the	0.101	0.155	**0.833**	0.054	0.17	0.114		

community to deliver health notifications through WeChat								
**Social Interaction**								
Through WeChat official account and Moments, I can keep abreast of the latest developments in public health events	0.314	0.15	0.052	**0.816**	0.139	0.16		
WeChat allows me to monitor and filter health-related rumors and fake news	0.224	0.15	0.049	**0.812**	0.053	0.065	3.70	0.90
WeChat provides timely and authoritative coverage of public health issues and health policy	0.136	0.151	0.068	**0.81**	0.124	0.073		
**Surveillance Need**								
To show my care to my family and friends by sharing meaningful health information on WeChat	0.179	0.191	0.188	0.093	**0.79**	0.122		
To filter health-related misinformation and offer high quality information for WeChat friends and groups	0.192	0.181	0.193	0.168	**0.748**	0.117	3.79	0.86
To build and consolidate relationships by sharing important health information	0.209	0.278	0.225	0.078	**0.72**	0.1		
**Self-Agency Building**								
I feel empowered to share health information and to give health-related advice to WeChat friends and groups	0.127	0.134	0.191	0.133	0.148	**0.891**		
							3.64	0.95
To express personal stance on health issues makes me feel satisfied	0.154	0.164	0.175	0.128	0.137	**0.889**		
Eigenvalue	3.19	2.733	2.487	2.254	2.084	1.783		
Variance explained (%)	15.951	13.667	12.437	11.269	10.422	8.914		
Cronbach's alpha	0.846	0.739	0.706	0.84	0.806	0.898		
